# Factors affecting waiting times to enter opioid use disorder treatment in the United States with a particular focus on potential biological sex disparity

**DOI:** 10.1002/pcn5.70048

**Published:** 2024-12-27

**Authors:** Stanley Nkemjika, Kenneth Oforeh, Connie Olwit, Olaniyi Olayinka, Terence Tumenta

**Affiliations:** ^1^ Department of Psychiatry and Human Behavior Thomas Jefferson University Philadelphia Pennsylvania USA; ^2^ Department of Psychiatry University of Pittsburgh Medical Center Pittsburgh Pennsylvania USA; ^3^ Department of Nursing Makerere University Kampala Uganda; ^4^ College of Nursing Augusta University Augusta Georgia USA; ^5^ Department of Psychiatry University of Texas Health Science Center Houston Texas USA; ^6^ Department of Psychiatry Yale School of Medicine New Haven Connecticut USA

**Keywords:** disparities, opioid use disorder, sex or biological sex difference, sociodemographic attributes, wait time

## Abstract

**Aim:**

Opioid use disorder (OUD) is the problematic use of licit or illicit opioids. Thus far, the literature on biological sex differences in accessing treatment is scarce. Hence, we hypothesize that biological sex has a moderating effect on OUD treatment accessibility. We aim to explore biological sex differences in treatment disparities and its role in wait time to enter OUD treatment.

**Method:**

Using the 2018 Treatment Episode Data Set Discharge, the national sample representative of OUD patients in the substance use disorder treatment facilities within the 50 states of the United States was 382,547. Univariate and multivariable logistic analysis of the independent variables and other covariates with the dependent variables were explored to estimate the adjusted odds relationship.

**Results:**

Medications for OUD (MOUD) use among males was significantly different, with waiting 1–7 days (adjusted odds ratio [AOR] = 1.300, 95% confidence interval [CI] = 1.208–1.399) and >7 days (AOR = 0.676, 95% CI = 0.600–0.763) to enter OUD treatment compared to waiting less than a day. For females, MOUD was significantly different, with waiting 1–7 days (AOR = 1.366, 95% CI = 1.244–1.499) and >7 days (AOR = 0.834, 95% CI = 0.721–0.965) to enter OUD treatment compared to waiting less than a day.

**Conclusion:**

Both females and males who received MOUD had lower odds of waiting >7 days to enter treatment than those who received no MOUD. The findings of this study will ensure equity in allocating resources to both males and females.

## INTRODUCTION

Opioid use disorder (OUD) is the problematic use of licit or illicit opioids, and the symptoms may manifest as intense cravings for opioids and a loss of control of their use.^1^ Opioids have naturally existed since the 1800s. However, since the 1990s, opioid use and related deaths have steadily risen; in 2017, the opioid crisis was announced as a public health concern.^2^ The surge in opioid use in the 1990s has been linked to the aggressive marketing strategy and prescribing practices by biopharmaceutical companies and healthcare providers, respectively.^3^ In addition to prescription opioids, illegal opioids have significantly contributed to thousands of opioid‐related fatalities.^4^

Thus far, much research on OUD has been conducted, including biological sex‐based studies, seemingly showing mixed results.^5^ According to the National Survey on Drug Use and Health and the Research Abuse, Diversion and Addiction‐Related Surveillance System, prescription‐related OUD is more common among men than women.^6^ Conversely, other studies suggest a higher percentage of females using prescription medications compared with males.^7^ Studies of biological sex differences in clinical settings show that women report higher substance/opioid cravings and higher rates of medical comorbidity compared with men.^8^ Additionally, women have been shown to progress rapidly from opioid initiation to more severe disease.^6^ However, comorbid substance use with opioids and cannabis was more prevalent among men. In contrast, stimulant and sedative use was more prevalent in women.^6^

Addressing the distinct challenges that men and women encounter when seeking care requires an understanding of wait time differences in OUD treatment by biological sex. Biological sex disparities significantly impact the ways in which people enter treatment and their experiences during treatment. According to our previous research on a related topic, we portrayed that women were disproportionately impacted by sociocultural issues such as stigma and caregiving obligations, which may result in prolonged wait periods to get admitted into treatment services.^9^ Hence, recognizing these differences can help highlight systemic problems in healthcare that could delay or prevent easy and quick access to necessary treatment modalities, which can guide improvements in practice and policy meant to advance equity in healthcare access. Notably, analyzing particular wait times, such as 1–7 days and more prolonged than 7 days, offers reasonable critical insights regarding the urgency and accessibility of OUD treatment based on the Treatment Episodes Data Set (TEDS) questionnaire designed by the Substance Abuse and Mental Health Services Administration (SAMHSA).^10^ Research evidence showed that more extended wait periods are linked to worse adverse outcomes, such as a higher chance of overdosing and relapsing.^11^ For instance, Nkemjika et al. found that individuals waiting more than a week to begin treatment were significantly less likely to engage in ongoing care, underscoring the importance of these timeframes in evaluating treatment accessibility.^9^

Despite the documented success in reducing OUD morbidity and mortality following treatment,^12^ there remains a considerable concern about accessing OUD treatment in the United States.^13^, ^14^ Supportive evidence suggests that among people seeking substance use disorder (SUD) treatment, the most common reason for not initiating treatment was a long waiting time.^15^ Although there are a few studies on disparities and barriers in accessing treatment for SUD,^16^, ^17^, ^18^ literature on biological sex differences in accessing treatment is scarce. Thus, the rationale for comparing wait times by biological sex in OUD treatment lies in the potential to enhance our understanding of healthcare disparities and improve access to care. Ultimately, this study can improve health outcomes for all individuals affected by OUD, recognizing that timely access to treatment is a critical component of effective intervention. Hence, we aimed to explore biological sex differences in treatment disparities and their role in waiting to enter OUD treatment. We hypothesize that biological sex has a moderating effect on OUD treatment accessibility.

## METHODOLOGY

### Data and samples

The TEDS used in this study is a cross‐sectional format data collection system consisting of the admission data set and the discharge‐specific data set. TEDS is estimated to include 83% of all eligible drug or alcohol treatment admissions in the United States. Data on OUD were obtained from the TEDS Discharge (TEDS‐D), a national data system of annual admissions and discharge to substance use disorder treatment facilities administered by SAMHSA. Facilities reporting OUD treatment from the TEDS‐D are those that received state opioid use agency funds (including Federal Block Grant funds) for the provision of OUD treatment. It is noteworthy that each observation in the OUD sample for this national data represents an OUD treatment episode and discharge records. The sample included records on participants aged 12 years or older. Notable information on admission demographics (e.g., age, sex, race/ethnicity, employment status, etc.) and OUD characteristics (e.g., opioid abuse, opioid dependence, intravenous drug use, use of opioid medications for treatment) were also included. SAMHSA ensures that the publicly available data complies with the Health Insurance Portability and Accountability Act.

### Analytical sample and inclusion criteria

The full sample of 2018 TEDS‐D (*n* = 382,547) was the total representation of OUD patients that utilized SUD treatment facilities within the 50 states of the United States. The data set was collected as such that all OUD diagnoses and treatment types had uniform acceptance criteria for treatment acceptance. Furthermore, we utilized the data based on the initial acceptance at baseline (first time in OUD treatment) to identify factors associated with this first treatment admission in relation to waiting time. Hence, analyses were inclusive of all clients based on their diagnosis, managed in all the facilities, and reported by the TEDS‐D in which clients were 12 years or older and were admitted for OUD treatment.

### Measures

#### Independent variable

Medication for OUD (MOUD) was the independent variable. It represents information on the use of medications like methadone, buprenorphine, and/or naltrexone as part of client's treatment plan. Respondents responded “Yes” or “No” for MOUD.

#### Dependent variables

Respondents were asked to describe service and system factors with regard to their admission, treatment, and discharge. Our main independent variable of concern was “days of wait time.” The number of days waiting to enter treatment indicated the number of days from the first contact or request for service until the client was admitted and/or the first clinical service was provided to the client. It did not include time delays caused by client unavailability or client failure to meet any requirement or obligation. Response was categorized in the data set as 0, 1, 2, 3, or 4 representing 0, 1–7, 8–14, 15–30, or 31 or more days, respectively. We recategorized these as 0, 1–7, and >7 days due to the few responses in the other cells following subgroup analysis.

#### Explanatory variables

Based on the literature review, individual demographics included client age, marital status, and education (years in school). Respondents also reported psychosocial characteristics, including employment status (full‐time, part‐time, unemployed and seeking employment, unemployed and not seeking employment, or not in the labor force), homelessness status (stable housing or homeless), racial disposition (dichotomously re‐categorized as Alaskan Natives, Black/African Americans, Asian/Pacific Islanders, Whites, and Others [included American Indian, Asian, other single race, two or more races and Native Hawaiian or other Pacific Islanders]). The “Other” category represented the length of stay in treatment, intravenous drug use, geographic regions of treatment facilities, primary source of payment for treatment anticipated at the time of admission, principal source of financial support, and type of treatment settings recategorized as detoxification, rehabilitation, and ambulatory services. The interaction effects of biological sex were also explored between waiting times and MOUD with other covariates.

### Statistical analysis

All forms of OUD treatment and admissions were included in the analysis. Data measured on a continuous scale were summarized with means, standard deviations, ranges, and medians. Categorical data were summarized with counts and percentages. The appropriate *χ* ^2^ tests were utilized to assess the relationships and differences between groups as respective statistically significant estimates were obtained. Univariate logistic analysis of the independent variables and other covariates with the dependent variables were explored to estimate the unadjusted odds ratio relationship. To test the association between explanatory variables and MOUD, multivariable logistic regression models were created for all independent study variable relationships with the dependent variable to estimate the true effect of each outcome. Hence, polytomous logistic regression models were used to assess the impact of predictive variables that were statistically significantly associated (P‐value < 0.05) with both exposure and MOUD. We also adjusted for confounding by including covariates based on evidence from the literature. Subanalysis by biological sex was conducted following a statistically significant interaction term. The TEDS‐D survey design did not take into account weighted variables and the possibility of missing values in some variables from respondents. All analyses and graphical presentations were performed using SAS software 9.4 (SAS Institute) for advanced statistical analysis, which handles complex design models. For all analyses in this study, a P‐value of <0.05 was considered statistically significant.

### Ethical consideration

As TEDS data are publicly available without subject identification as the study has an Institutional Review Board exempt status.

## RESULTS

### Sociodemographic characteristics of OUD treatment among 2018 US population

Table 1 shows the sociodemographic characteristics of respondents who were admitted and treated for OUD in the United States for the year 2018. The majority (male [M] = 76.69%, female [F] = 77.05%) of OUD treatment respondents were between the ages of 25 and 49 years, non‐Hispanic White (M = 78.01%, F = 84.16%). In terms of educational status, 4.20% (males) and 5.34% (females) of OUD treatment individuals had a 4‐year college degree or more, and most of the participants had a 12th grade level of education with a prevalence rate of 54.70% for males and 46.86% for females. Approximately, 48% of male and 52.18% of female participants were unemployed and 16.54% of male and 9.24% of female respondents were not currently in full‐time employment status. The vast majority of OUD patients (M = 73.61%, F = 65.43%) had never been married. Approximately 93% of both males and females had never been arrested within 30 days before initiating treatment. With regards to source of income, most (M = 44.95%, F = 46.21%) of the OUD patients had no source of income, while those with salary/wages (M = 26.38%, F = 19.77%) had the second highest prevalence, followed by those receiving public assistance (M = 7.13%, F = 9.59%), and a few respondents (M = 6.06%, F = 7.44%) relied on a retirement pension or disability benefit as their primary source of income. Most of the respondents receiving treatment reported getting onto the OUD treatment program in less than a day, while 28.22% of males and 26.09% of females reported waiting between 1 and 7 days to get onto a treatment program. Our study population reported that 64.47% of males and 72.79% of females were managed in ambulatory settings. In terms of geographical regions, the south (M = 35.91, F = 43.08%) had the largest frequency, followed by the northeast region (M = 35.85%, F = 25.86%). In terms of diagnostic and statistical manual of mental disorders IV classification, the population had more of patients with opioid abuse diagnosis (∼96%). See Table 1 for more details.

**Table 1:** Sociodemographic descriptive and clinical attributes of the OUD treatment population by biological sex (*N* = 382,498).

Demographic characteristics	Males (*N* = 226,081)		Females (*N* = 156,417)	
	*N*	%	*N*	%
Age (years)				
12–24	22,101	9.78	19,174	12.26
25–49	172,813	76.69	120,519	77.05
>50	31,167	13.79	16,724	10.69
Race				
Alaskan Native/American Indian	2015	0.93	1990	1.31
Asian/Pacific Islander/Hawaiian Native	1723	0.79	714	0.47
Black/African American	29,122	13.40	15,367	10.14
White	16,9475	78.01	127,587	84.16
Other single race/two or more races	14,920	6.87	5945	3.92
Education				
<1 school grade–8 grade	7710	3.78	4904	3.49
Grades 9–11	40,280	19.77	28,024	19.93
Grade 12	111,468	54.70	65,878	46.86
1–3 years of college	35,748	17.54	34,281	24.38
>4 years	8558	4.20	7506	5.34
Employment status				
Full‐time	34,020	16.54	13,080	9.24
Part‐time	13,449	6.54	11,035	7.80
Unemployed	98,566	47.92	73,862	52.18
Not in labor force	59,638	29.00	43,579	30.79
Marital status				
Never married	14,4787	73.61	90,366	65.43
Currently married	21,590	10.98	16,814	12.17
Divorced/separated/widowed	30,330	15.42	30,927	22.39
Health insurance				
Private	6387	6.25	3581	4.75
Medicaid	60,456	59.17	52,022	68.99
Medicare, other (TRICARE, etc.)	8142	7.97	5343	7.09
None	27,182	26.61	14,462	19.18
Services waiting time				
0 days	63,210	62.44	43,226	63.63
1–7 days	28,569	28.22	17,724	26.09
>1 week	9450	9.34	6986	10.28
Arrest in past 30 days prior to admission				
None	193,632	92.70	135,970	92.66
Once	11,851	5.67	8531	5.81
Two or more times	3400	1.63	2237	1.52
Source of income				
Wages/salary	30,431	26.38	15,716	19.77
Public assistance	8223	7.13	7624	9.59
Retirement/pension, disability	6987	6.06	5913	7.44
Other	17,869	15.49	13,509	16.99
None	51,852	44.95	36,730	46.21
Living arrangement				
Homeless	28,377	14.25	14,476	10.54
Dependent living	32,816	16.48	23,368	17.01
Independent living	137,925	69.27	99,499	72.45
Type of treatment service				
Detox (hospital inpatient, residential)	42,039	18.59	20,324	12.99
Rehab (both short and long term)	38,292	16.94	22,243	14.22
Ambulatory, intensive outpatient	145,750	64.47	113,850	72.79
Primary payment source				
Self‐pay	3919	3.84	2331	3.09
Private insurance	4885	4.78	2956	3.92
Medicare	1458	1.43	1343	1.78
Medicaid	61,685	60.39	53,125	70.39
Other government payments	24,015	23.51	11,435	15.15
No charge (free, charity, teaching, etc.)	2148	2.10	914	1.21
Other	4037	3.95	3367	4.46
Census region				
US territories	638	0.28	86	0.05
Northeast	81,055	35.85	40,450	25.86
Midwest	35,105	15.53	27,253	17.42
South	81,187	35.91	67,377	43.08
West	28,096	12.43	21,251	13.59
Heroine reported on admission				
Yes	15,7806	69.80	97,279	62.19
No	68,275	30.20	59,138	37.81
Other opiate reported on admission				
Yes	62,110	27.47	54,826	35.05
No	163,971	72.53	101,591	64.95
Intravenous drug use				
Yes	111,842	81.41	73,800	80.17
No	25,540	18.59	18,252	19.83
Medication‐assisted therapy				
Yes	61,341	28.89	45,713	31.04
No	151,001	71.11	101,540	68.96
Opioid use disorder				
Opioid abuse	216,634	95.82	149,499	95.58
Opioid dependence	9447	4.18	6918	4.42

### Differences by biological sex for days waiting to enter treatment by medication‐assisted opioid therapy and other sociodemographic attributes

Following the test for moderating effect of biological sex differences among respondents, which was statistically significant, Table 2 shows the adjusted association of days waiting to enter treatment with MOUD and other covariates by biological sex.

**Table 2:** Biological sex differences for waiting time to enter OUD treatment for 2018 US population.

Sociodemographic characteristics	Waiting times			
	Male		Female	
	1–7 days	>1 week	1–7 days	>1 week
	AOR (95% CI)	AOR (95% CI)	AOR (95% CI)	AOR (95% CI)
Length of stay (days)	1.009 (1.006–1.012)**	1.027 (1.022–1.032)**	1.006 (1.002–1.010)*	1.014 (1.008–1.020)**
Age (years)				
12–24	Ref			
25–49	0.941 (0.848–1.046)	0.979 (0.844–1.135)	1.079 (0.958–1.216)	0.950 (0.807–1.119)
>50	0.819 (0.703–0.953)*	0.650 (0.511–0.826)*	0.911 (0.742–1.120)	0.671 (0.483–0.933)*
Race				
Alaskan Native/American Indian	1.273 (0.870–1.864)	1.369 (0.860–2.179)	0.808 (0.498–1.309)	1.604 (0.943–2.729)
Asian/Pacific Islander/Hawaiian Native	0.932 (0.700–1.239)	0.436 (0.257–0.739)*	0.948 (0.619–1.452)	0.779 (0.371–1.634)
Black/African American	Ref			
White	1.073 (0.960–1.199)	1.256 (1.053–1.499)*	1.160 (0.991–1.359)	1.683 (1.285–2.203)*
Other single race/two or more races	0.774 (0.588–1.017)	1.026 (0.745–1.412)	1.020 (0.753–1.383)	1.401 (0.927–2.117)
Education				
<1 school grade–8 grade	Ref			
Grades 9–11	1.031 (0.854–1.245)	1.428 (1.058–1.927)*	1.123 (0.866–1.458)	0.855 (0.606–1.207)
Grade 12	1.058 (0.883–1.267)	1.238 (0.927–1.655)	1.208 (0.940–1.552)	0.928 (0.668–1.289)
1–3 years of college	0.982 (0.807–1.195)	1.247 (0.917–1.695)	1.076 (0.828–1.398)	0.799 (0.566–1.127)
>4 years	1.286 (1.025–1.615)*	1.574 (1.107–2.238)*	1.571 (1.171–2.107)*	1.277 (0.868–1.877)
Employment status				
Full‐time	1.109 (0.966–1.273)	1.290 (1.060–1.569)*	1.032 (0.841–1.268)	1.284 (0.972–1.697)
Part‐time	1.199 (1.020–1.410)*	1.389 (1.099–1.757)*	1.399 (1.141–1.716)*	1.412 (1.065–1.873)*
Unemployed	Ref			
Not in the labor force	1.860 (1.723–2.007)**	1.731 (1.550–1.934)**	1.611 (1.470–1.766)**	1.152 (1.008–1.316)*
Marital status				
Never married	Ref			
Currently married	1.045 (0.930–1.174)	1.261 (1.073–1.482)*	0.957 (0.835–1.097)	1.022 (0.845–1.236)
Divorced/separated/widowed	1.037 (0.943–1.141)	1.250 (1.097–1.426)*	1.039 (0.934–1.157)	1.093 (0.942–1.269)
Health insurance				
Private	1.043 (0.886–1.228)	1.315 (1.056–1.636)*	1.139 (0.910–1.426)	1.398 (1.038–1.882)*
Medicaid	1.592 (1.446–1.752)**	1.611 (1.407–1.843)**	1.619 (1.434–1.829)**	1.532 (1.294–1.813)**
Medicare, other (TRICARE, etc.)	0.365 (0.316–0.422)**	0.678 (0.561–0.820)*8	0.471 (0.387–0.572)**	0.764 (0.591–0.987)*
None	Ref			
Source of income				
Wages/salary	Ref			
Public assistance	0.659 (0.554–0.785)**	0.736 (0.558–0.970)*	0.824 (0.669–1.013)	1.097 (0.820–1.469)
Retirement/pension, disability	0.662 (0.551–0.795)**	0.630 (0.470–0.846)*	0.677 (0.537–0.854)*	0.915 (0.649–1.289)
Other	1.479 (1.280–1.709)**	1.736 (1.413–2.132)**	1.371 (1.132–1.695)*	1.475 (1.128–1.928)*
None	0.713 (0.627–0.809)**	0.924 (0.772–1.106)	0.841 (0.707–1.001)	0.977 (0.770–1.241)
Type of treatment service				
Detox (hospital inpatient, residential)	0.818 (0.720–0.931)*	0.775 (0.617–0.972)*	0.847 (0.706–1.016)	0.840 (0.623–1.131)
Rehab (both short and long term)	1.013 (0.927–1.108)	1.763 (1.570–1.981)**	1.217 (1.086–1.363)*	1.958 (1.695–2.262)**
Ambulatory, intensive outpatient	Ref			
Primary payment source				
Self‐pay	2.591 (2.239–2.999)**	1.308 (1.060–1.613)*	2.596 (2.157–3.125)**	1.475 (1.133–1.919)*
Private insurance	2.794 (2.326–3.357)**	0.813 (0.606–1.092)	2.403 (1.874–3.081)**	0.599 (0.399–0.900)*
Medicare	1.422 (0.890–2.274)	0.590 (0.253–1.376)	0.547 (0.308–0.972)*	0.914 (0.471–1.776)
Medicaid	1.091 (0.987–1.206)	0.811 (0.704–0.935)*	0.989 (0.875–1.117)	0.821 (0.692–0.975)*
Other government payments	Ref			
No charge (free, charity, teaching, etc.)	0.951 (0.737–1.227)	1.151 (0.865–1.532)	1.841 (1.367–2.480)**	1.726 (1.237–2.408)*
Other	1.107 (0.907–1.351)	0.751 (0.582–0.968)*	1.029 (0.809–1.309)	0.954 (0.717–1.268)
Census region				
US territories	0.324 (0.189–0.557)**	0.762 (0.421–1.381)	0.027 (0.003–0.206)*	__
Northeast	Ref			
Midwest	0.069 (0.059–0.081)**	0.321 (0.270–0.383)**	0.064 (0.054–0.076)**	0.300 (0.247–0.365)**
South	0.118 (0.106–0.131)**	0.187 (0.159–0.221)**	0.128 (0.112–0.148)**	0.266 (0.218–0.328)**
West	0.133 (0.119–0.148)**	0.515 (0.452–0.587)**	0.175 (0.154–0.198)**	0.511 (0.433–0.603)**
Intravenous drug use				
Yes	0.860 (0.787–0.939)*	0.865 (0.766–0.978)*	0.778 (0.696–0.870)**	0.707 (0.610–0.820)**
No	Ref			
Medication‐assisted opioid therapy				
Yes	1.300 (1.208–1.399)**	0.676 (0.600–0.763)**	1.366 (1.244–1.499)**	0.834 (0.721–0.965)*
No	Ref			

Abbreviations: AOR, adjusted odds ratio; CI, confidence interval.

#### The length of stay in treatment and waiting times

There was a statistically significant association between the length of stay in treatment and waiting times, with males experiencing longer wait times for both 1–7 days (adjusted odds ratio [AOR] = 1.009; 95% confidence interval [CI] = 1.006–1.012) and >7 days (AOR = 1.027, 95% CI = 1.022–1.032) compared to 0 days. Similarly, females experienced longer wait times for both 1–7 days (AOR = 1.006, 95% CI = 1.002–1.010) and >7 days (AOR = 1.014, 95% CI = 1.008–1.020) compared to 0 days, but the odds for females waiting longer than males were slightly lower.

#### Age and waiting times

For males older than 50 years, there was a shorter waiting time to enter treatment for both 1–7 days (AOR = 0.819, 95% CI = 0.703–0.953) and >7 days (AOR = 0.650, 95% CI = 0.511–0.826). Conversely, females older than 50 years had shorter waiting times, but only for waiting >7 days (AOR = 0.671, 95% CI = 0.483–0.933).

#### Race/ethnicity and waiting times

Asian/Pacific Islander males had significantly lower odds of waiting more than a week to enter treatment compared to Black/African American males (AOR = 0.436, 95% CI = 0.257–0.739). Non‐Hispanic White males had significantly higher odds of waiting >7 days compared to Black males (AOR = 1.256, 95% CI = 1.053–1.499). At the same time, non‐Hispanic White females had 1.683 times greater odds of waiting more than a week compared to Black females (AOR = 1.683, 95% CI = 1.285–2.203). There were no significant differences for “Other” races.

#### Educational attainment and waiting times

Males with a college degree had significantly higher odds of waiting longer to enter treatment for both 1–7 days (AOR = 1.286, 95% CI = 1.025–1.615) and >7 days (AOR = 1.574, 95% CI = 1.107–2.238). Similarly, females with a college degree had higher odds of waiting 1–7 days (AOR = 1.571, 95% CI = 1.171–2.107), but the effect was more substantial for females with a college degree than males.

#### Employment status and waiting times

Employment status significantly impacted waiting times for males. Full‐time employment was associated with increased odds of waiting >7 days compared to unemployment (AOR = 1.290, 95% CI = 1.060–1.596). Part‐time employment was also associated with increased odds of waiting, particularly for >7 days (AOR = 1.389, 95% CI = 1.099–1.757). Males not in the labor force had the highest odds of waiting compared to the unemployed group, with AOR = 1.860 (95% CI = 1.723–2.007) for 1–7 days and AOR = 1.731 (95% CI = 1.550–1.934) for >7 days. Similarly, for females, part‐time employment was associated with significantly higher odds of waiting both 1–7 days (AOR = 1.399, 95% CI = 1.141–1.716) and >7 days (AOR = 1.412, 95% CI = 1.065–1.873). Females not in the labor force also had higher odds of waiting compared to the unemployed group (AOR = 1.611, 95% CI = 1.470–1.766 for 1–7 days; AOR = 1.152, 95% CI = 1.008–1.316 for >7 days).

#### Insurance coverage and waiting times

Among males, Medicaid recipients had significantly higher odds of waiting both 1–7 days (AOR = 1.592, 95% CI = 1.446–1.752) and >7 days (AOR = 1.611, 95% CI = 1.407–1.843). In contrast, Medicare recipients had lower odds of waiting for both 1–7 days (AOR = 0.365, 95% CI = 0.316–0.422) and >7 days (AOR = 0.678, 95% CI = 0.561–0.820). Private insurance was also significantly associated with longer wait times for >7 days (AOR = 1.315, 95% CI = 1.056–1.636). For females, Medicaid coverage also increased the odds of waiting for both 1–7 days (AOR = 1.619, 95% CI = 1.434–1.829) and >7 days (AOR = 1.532, 95% CI = 1.294–1.813), while Medicare was associated with shorter wait times for both periods (AOR = 0.471, 95% CI = 0.387–0.572 for 1–7 days; AOR = 0.764, 95% CI = 0.591–0.987 for >7 days). Private insurance was linked to longer wait times for >7 days (AOR = 1.398, 95% CI = 1.038–1.882).

#### Income source and waiting times

Compared to wages/salary, retired/pensioned males had lower odds of waiting 1–7 days (AOR = 0.662, 95% CI = 0.551–0.795) and >7 days (AOR = 0.630, 95% CI = 0.470–0.846). Other income sources among males were associated with significantly higher odds of waiting for both periods (AOR = 1.479, 95% CI = 1.280–1.709 for 1–7 days and AOR = 1.736, 95% CI = 1.413–2.132 for >7 days). Similarly, for females, other income sources were associated with higher odds of waiting both 1–7 days (AOR = 1.371, 95% CI = 1.132–1.695) and >7 days (AOR = 1.475, 95% CI = 1.128–1.928). Retired/pensioned females showed lower odds of waiting 1–7 days (AOR = 0.659, 95% CI = 0.554–0.785).

#### Geographical location and waiting times

Both males and females exhibited significant regional differences in waiting times. Treatment in the Midwest, South, West, and other US territories showed significantly shorter wait times compared to the Northeast:

- Midwest: AOR = 0.069 (95% CI = 0.059–0.081) for 1–7 days, AOR = 0.321 (95% CI = 0.270–0.383) for >7 days (males), and AOR = 0.064 (95% CI = 0.054–0.076) for 1–7 days, AOR = 0.300 (95% CI = 0.247–0.365) for >7 days (females)
- South: AOR = 0.118 (95% CI = 0.106–0.131) for 1–7 days, AOR = 0.187 (95% CI = 0.159–0.221) for >7 days (males), and AOR = 0.128 (95% CI = 0.112–0.148) for 1–7 days, AOR = 0.266 (95% CI = 0.266–0.328) for >7 days (females)
- West: AOR = 0.133 (95% CI = 0.119–0.148) for 1–7 days, AOR = 0.515 (95% CI = 0.452–0.587) for >7 days (males), and AOR = 0.175 (95% CI = 0.154–0.198) for 1–7 days, AOR = 0.511 (95% CI = 0.433–0.603) for >7 days (females).

#### Treatment service type and waiting times

Among males, Detox services were associated with shorter wait times for both 1–7 days (AOR = 0.818, 95% CI = 0.720–0.931) and >7 days (AOR = 0.775, 95% CI = 0.617–0.972), while rehabilitation services showed longer wait times for >7 days (AOR = 1.763, 95% CI = 1.570–1.981). Similarly, rehabilitation services for females were associated with significantly longer wait times for both 1–7 days (AOR = 1.217, 95% CI = 1.086–1.363) and >7 days (AOR = 1.958, 95% CI = 1.695–2.262).

#### Drug use and waiting times

Intravenous drug use among males was associated with shorter waiting times for both 1–7 days (AOR = 0.860, 95% CI = 0.787–0.939) and >7 days (AOR = 0.865, 95% CI = 0.766–0.978). Similarly, intravenous drug use among females was associated with shorter waiting times for both 1–7 days (AOR = 0.778, 95% CI = 0.696–0.870) and >7 days (AOR = 0.707, 95% CI = 0.610–0.820).

#### MOUD and waiting times

Among males, MOUD use increased the odds of waiting 1–7 days (AOR = 1.300, 95% CI = 1.208–1.399) but decreased the odds for waiting >7 days (AOR = 0.676, 95% CI = 0.600–0.763). For females, MOUD use was also associated with longer waiting times for 1–7 days (AOR = 1.366, 95% CI = 1.244–1.499) but shorter waiting times for >7 days (AOR = 0.834, 95% CI = 0.721–0.965).

## DISCUSSION

Our research focuses on two biological sex differences: sociodemographic characteristics and the time it takes to enter OUD treatment. The results showed that the 12–24 years age range had a higher proportion of females, while the over‐50 years age range was dominated by men. The majority of women paid for treatment services with Medicaid, whereas the majority of men either did not have insurance or paid with cash or private insurance. The sociodemographic attributes of study participants are similar to what has been reported in the scientific literature. For instance, the majority of OUD treatment populations are male, aged 18–25 years, use commercial insurance, and are non‐Hispanic White.^19^ In terms of educational status, women had a higher prevalence of college education than men, as the majority had a 12th‐grade education. However, while men were more likely to have full‐time employment, females were more likely to be on public assistance and to work part‐time. In addition, men in the northeastern part of the country are more likely than women to have never married, been homeless, or received detox or rehab treatment. Females reported using more ambulatory intensive outpatient services, being divorced, and living on their own.

For both sexes, having a college degree was associated with a greater likelihood of waiting between 1 and 7 days to begin treatment. Males who finished grades 9–11 of schooling were noted to wait more than 1 week to enter OUD treatment compared to females with a similar level of education. Male and female patients were more likely to wait between 1 and 7 days and longer than 7 days to begin OUD treatment if they had a full‐time or part‐time job, as opposed to being unemployed. Further investigation revealed that females were slightly more likely than males to obtain MOUD. Yang and colleagues used the TEDS‐A data set to examine differences in opioid treatment receipt and treatment entry time in the United States between 2014 and 2017. Their study finding was similar to our study as the probability of women receiving MOUD was slightly higher than that of men. However, they found no statistically significant difference between men and women in the amount of time it took to enter treatment.^20^

Furthermore, we found a similar trend in the length of stay in treatment for both men and women, although females had slightly lesser odds of days waiting to enter treatment. We provide a brief context of the population that Marsha and colleagues examined in Los Angeles, which found that women spent more time than men waiting to enter treatment even though they remained in treatment longer. The authors found differences at the racial level as African American, Hispanic, and Other females were at a higher risk of shorter treatment duration than non‐Hispanic women and men.^21^ Khachikian et al.'s study found that factors such as being Hispanic, having a co‐occurring mental illness, having children under the age of 18 living in the home, having a history of prior treatment, and having received methadone treatment and counseling services significantly contributed to the longer retention in treatment for pregnant women.^22^

Marital status did not show statistically significant differences in waiting time for both sexes. However, men were more likely to wait longer than seven days to begin treatment if they were married or divorced. For both sexes, having some kind of health insurance, especially Medicaid or private insurance, at admission was linked to a higher likelihood of waiting between 1 and 7 days and longer to enter treatment. On the other hand, when compared to Medicaid‐paid services and other forms of compensation, self‐payment and payment through private insurance were associated with a shorter wait time to enter treatment. The locations of treatment facilities remained significantly distinct across all regions for both sexes. When we separated the kinds of treatment services, we found that male participants had shorter wait times for detox than female participants did for rehabilitation services. The study did not find any statistically significant relationship between the age of the patients and biological sex.

A breakdown by age group showed that males and females older than 50 years had a shorter waiting time to enter treatment. Regarding race/ethnicity, Asian/Pacific Islander males and females had lesser odds of waiting more than a week to enter treatment. In comparison, non‐Hispanic White males and females had greater odds of waiting more than a week to enter treatment. Other studies have found racial disparities in completing treatment plans at discharge for Hispanic women compared to male and non‐Hispanic White patients.^23^

Understanding the disparities in treatment services and wait times for OUD between biological sexes is crucial for enhancing care delivery. Our analysis also revealed significant differences in the types of services utilized and the duration of waiting periods experienced by male and female patients. For males, detoxification services showed a notably lower AOR for waiting times of 1–7 days (AOR = 0.818) and more than 7 days (AOR = 0.775). Conversely, rehabilitation services resulted in a higher likelihood of longer wait times of over 7 days, indicating a potential bottleneck in rehabilitation services for males, which may delay access to necessary care and adversely affect treatment outcomes. For female patients, the scenario is similarly nuanced as rehabilitation services also presented higher odds ratios for waiting times for 1–7 days (AORs = 1.217) and more than 7 days (AORs = 2.262). This suggests that males accessing detox services are more likely to experience shorter wait times compared to other treatment options. At the same time, females may face substantial delays when seeking rehabilitation services, a situation that can exacerbate the severity of their condition.

Furthermore, intravenous drug use was associated with shorter waiting times for both biological sex, which suggests expedited access to treatment, possibly due to heightened urgency in their cases. Similarly, MOUD usage in our study also illustrated significant biological sex differences in wait times as males (AOR = 1.300) and females (AOR = 1.366) experienced faster access to treatment within 1–7 days. Hence, while both sexes have expedited access, females are less likely to wait as long as their male counterparts in certain circumstances. Therefore, these subtle biological sex disparities portray the intricacies that can affect wait times in OUD care and access to treatment. Notably, these differences could be explained by the healthcare system, societal expectations, stigma, and their influence on individuals' engagement with OUD treatment services. For instance, Nkemjika et al. highlighted that females may face additional challenges due to caregiving responsibilities or the stigma associated with substance use, which could lengthen wait times for necessary treatment modalities.^9^ Males, on the other hand, might experience less social stigma, but they still confront structural obstacles when trying to obtain some form of treatment, especially in rehabilitation clinics.^9^

### Strength and limitations

The use of the TEDS‐D data set has its strengths and limitations. The data set is a national representative of OUD admission and discharges in federally recognized treatment centers which depicts generalizability. The data set provides an opportunity to examine the treatment attributes of the OUD population in the United States. However, there are many demerits that may affect the outcome of the study. Notably, the cross‐sectional nature of the data set is flawed, as causality cannot be estimated. This limitation also cuts across the design of study variables during data collection. Lastly, the TEDS data set is administratively based that relies on self‐reported questionnaires and surveys, thus caution should be exercised concerning its interpretation.

## CONCLUSION

Among adults seeking OUD treatment admissions, wait times varied with MOUD use as there was early entry compared to >1 week wait time for both males and females. While both sexes shared specific trends in their waiting times to enter treatment, key differences included variations in the impact of age, education, race/ethnicity, and employment status. Males tended to experience longer wait times across various variables, particularly for employment status and education, while females showed a more robust association with educational attainment. Furthermore, treatment type and regional location have similar effects for both sexes, with both groups exhibiting significant regional disparities. Our study showed some differences with implications for clinical practice but did not find any statistically significant relationship with the age of the patients. Similarly, significant associations were reported across different sociodemographic attributes, but with a slight difference in the estimates. The findings of this study will ensure equity in allocating resources to both males and females. They will provide a platform for improved policy change and SUD treatment and care adjustment. Future studies should consider exploring the possible moderating effect of biological sex to estimate the actual relationship and bridge the literature gap.

## AUTHOR CONTRIBUTIONS

Stanley Nkemjika, Kenneth Oforeh, and Olaniyi Olayinka contributed to the conceptualization of the study. Stanley Nkemjika and Terence Tumenta conducted the literature review. Stanley Nkemjika, Connie Olwit, and Terence Tumenta abstracted and analyzed the data. Stanley Nkemjika and Olaniyi Olayinka drafted the main manuscript text. Stanley Nkemjika and Terence Tumenta critically reviewed, discussed, and modified the intellectual content of the article. All authors read and approved the final manuscript.

## CONFLICT OF INTEREST STATEMENT

The authors declare no conflicts of interest.

## ETHICS APPROVAL STATEMENT

Since this study was a secondary analysis and publicly available de‐identified data set, the study did not require any ethical approval.

## PATIENT CONSENT STATEMENT

N/A

## CLINICAL TRIAL REGISTRATION

N/A

## Data Availability

https://www.samhsa.gov/data/data-we-collect/teds-treatment-episode-data-set.
